# Liquid Chromatography-Tandem Mass Spectrometry Method for Detection and Quantification of Meloxicam and 5′-Carboxymeloxicam in Oral Fluid Samples

**DOI:** 10.3390/metabo13060755

**Published:** 2023-06-15

**Authors:** Gabriela Moraes Oliveira, Thiago José Dionísio, Viviane Silva Siqueira-Sandrin, Leticia Alves de Lima Ferrari, Bella Luna Colombini-Ishikiriama, Flávio Augusto Cardoso Faria, Carlos Ferreira Santos, Adriana Maria Calvo

**Affiliations:** 1 Hospital for Rehabilitation of Craniofacial Anomalies, University of São Paulo (HRAC/USP), Bauru 17012-900, SP, Brazil; gab.moraes@usp.br (G.M.O.); cfsantos@fob.usp.br (C.F.S.); 2 Department of Biological Sciences, Bauru School of Dentistry, University of São Paulo, Bauru 17012-901, SP, Brazil

**Keywords:** meloxicam, 5′-carboxymeloxicam, mass spectrometry, oral fluid

## Abstract

A sensitive, selective and particularly fast method of liquid chromatography-tandem mass spectrometry (LC-MS/MS) was developed and validated for the determination of meloxicam and its main metabolite, 5′-carboxymeloxicam, in oral fluid samples. Meloxicam and its major metabolite were separated using a Shim-Pack XR-ODS 75 L × 2.0 column and C18 pre-column at 40 °C using a mixture of methanol and 10 mM ammonium acetate (80:20, *v*/*v*) with an injection flow rate of 0.3 mL/min. The total time of the analytical run was 5 min. Sixteen volunteers had oral fluid samples collected sequentially before and after taking a meloxicam tablet (15 mg) for up to 96 h. With the concentrations obtained, the pharmacokinetic parameters were determined using the Phoenix WinNonlin software. The parameters evaluated for meloxicam and 5′-carboxymeloxicam in the oral fluid samples showed linearity, accuracy, precision, medium-quality control (MQC-78.12 ng/mL), high-quality control (HQC-156.25 ng/mL), lower limits of quantification (LLOQ-0.6103 ng/mL), low-quality control (LQC-2.44 ng/mL), stability and dilution. Prostaglandin E_2_ (PGE_2_) was also detected and quantified in the oral fluid samples, demonstrating the possibility of a pharmacokinetic/pharmacodynamic (PK/PD) study with this methodology. All the parameters evaluated in the validation of the methodology in the oral fluid samples proved to be stable and within the possible variations in each of the described parameters. Through the data presented, the possibility of a PK/PD study was demonstrated, detecting and quantifying meloxicam, its main metabolite and PGE_2_ in oral fluid samples using LC-MS/MS.

## 1. Introduction

**Figure 1:**
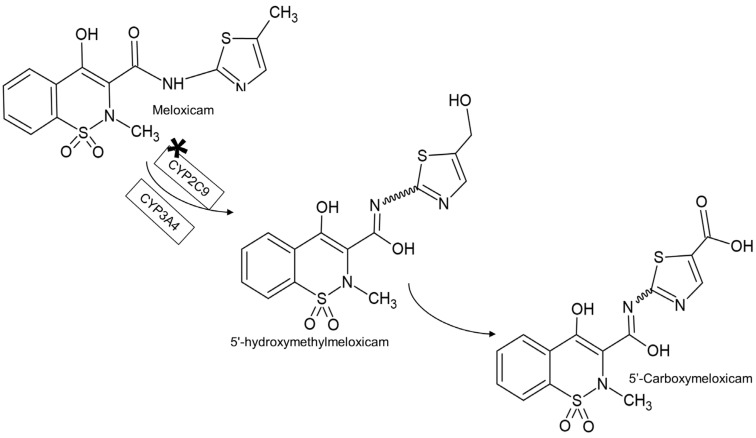
Meloxicam metabolism (* main enzyme responsible for the drug metabolism).

Meloxicam (4-hydroxy-2-methyl-N-(5-methyl-2-thiazolyl)-2H-1,2-benzothiazine-3-carboxamide-1,1-dioxide) is a non-steroidal anti-inflammatory drug (NSAID) that is preferentially selective for cyclooxygenase-2 (COX-2) [1,2]. It is used worldwide to control the signs and symptoms of inflammation, in particular musculoskeletal conditions, osteoarthritis and rheumatoid arthritis [3,4], in addition to being useful for postoperative pain control [5]. Meloxicam is highly metabolized, with cytochrome P4502C9 enzyme (CYP2C9) being the main enzyme responsible for the drug metabolism with a small contribution from cytochrome P4503A4 enzyme (CYP3A4). Almost 60% of the ingested dose is metabolized to its major metabolite, 5′-carboxymeloxicam, from an oxidation of liver cytochrome enzymes to an intermediate metabolite, 5′-hydroxymethylmeloxicam [6,7,8,9,10,11] (Figure 1).

Recent studies have demonstrated that allelic variations in *CYP2C9* are related to the increase in the adverse events induced by non-steroidal anti-inflammatory drugs (NSAIDs), including meloxicam. After a usual dose, variations in drug toxicity and inefficiency can be observed between different *CYP2C9* polymorphisms. Therefore, in order to avoid incorrect dosing, it is recommended to adjust the dose of meloxicam considering the demographic and genetic characteristics of the individuals [12,13,14,15].

Pharmacokinetic/pharmacodynamic (PK/PD) and physiologically cased pharmacokinetic (PBPK) studies have demonstrated the importance of evaluating the dose–concentration–response relationship, making it possible to describe and predict the effects of time resulting from a drug dose. The data obtained are essential for personalized prescription studies, being useful for guidance and dose adjustment in several clinical areas [16,17,18,19].

Since meloxicam selectively inhibits COX-2, prostaglandin E_2_ (PGE_2_) synthesis is also inhibited. In this sense, several studies have shown that PGE_2_ can be the main target of PK/PD studies. Changes in PGE_2_ concentrations, on the other hand, can be used to quantify the influence of COX-2 after the administration of the studied non-steroidal anti-inflammatory drugs (NSAIDs) [20,21].

NSAIDs are strongly bound to plasma proteins (97–99%); therefore, their concentration found in oral fluid corresponds to the free or unbound fraction of the drug circulating and distributed throughout the body, which is the most relevant data when assessing the pharmacological or toxic action of a drug [22,23,24,25].

Due to the numerous sample collections required for a pharmacokinetic study (up to 96 h), the use of alternative samples, such as oral fluid, has shown promise, even for NSAIDs [26,27,28,29,30,31]. Some analytical methodologies were developed in human plasma for the detection of meloxicam after the ingestion of a 7.5 mg tablet [32], 15 mg tablet [33] and transdermal application [34], but studies about the detection and determination of this drug in oral fluid samples have not been reported.

Although oral fluid is a great alternative as a matrix for PK/PD studies, one must consider that sample preparation for the detection of the analytes of interest is fundamental. Sample preparation is still one of the most complex and important steps for analytical analysis. Therefore, this step requires more time and, sometimes, more plastic materials. Such sample treatment in the pre-analytical phase aims to remove matrix components that may interfere with the final analysis and, thus, isolate the specific analyte, thus promoting a clean and specific sample [21,35,36].

Microextraction by packed sorbent (MEPS) has been shown to be a method with excellent results for the extraction of analytes in complex matrixes such as oral fluid. This methodology allows the removal of components from the matrix, reducing the interfering components and, thus, promoting a more accurate extraction of the analytes. MEPS is a miniaturization of solid-phase extraction (SPE) with a packed bed (1–2 mg). Although its applicability in oral fluid is recent, it has shown to be promising with some drugs [21,27,37,38]. 

In terms of evaluating the most used methods for analyte extraction such as liquid–liquid methods, MEPS demonstrates greater benefits, such as ease of use and process time. When compared to other methodologies, this device has advantages due to time savings. The various stages can be considered harmful to the results given the prolonged extraction and drying time, such as the non-ecological use of plastic material [26,28,29,30,31].

The present study was designed to develop a sensitive and reliable method for the detection and quantification of meloxicam, its main metabolite, 5′-carboxymeloxicam and PGE_2_, simultaneously, in oral fluid samples by LC-MS/MS. For that purpose, we evaluated the PK/PD parameters in volunteers within a period of 96 h after the ingestion of a 15 mg meloxicam oral tablet.

## 2. Experimental Design

This study was approved by the Research Ethics Committee of the Bauru School of Dentistry/University of São Paulo (CAAE 92312318.4.0000.5417) and was registered at Re-BEC (Brazilian Register of Clinical Trials RBR-38jcm9). All volunteers signed a consent form after being fully informed about the study content and procedures.

### 2.1. Chemicals and Reagents

Meloxicam (C14H13N3O4S2), meloxicam-D3 (internal standard (IS)—C14H10D3N3O4S2) and prostaglandin E_2_ (PGE_2_) (11,15-dihydroxy-9-oxoprost-5,13-dienoic acid) were purchased from Sigma-Aldrich^®^ (São Paulo, Brazil) (catalog Y0001080, 34109, P0409, respectively), and 5′-carboxymeloxicam (C14H11N3O6S2) was purchased from United Chemicals Instrumentos Científicos Ltda—ME (catalog SC207069—Santa Cruz). All standards used had >93% HPLC purity. Methanol (MeOH), ammonium acetate and other chemicals used in the tests were purchased from Merck (Hohenbrunn, Germany), all at a chromatographic grade, and water was obtained from a Milli-Q Plus purification system (Millipore, Belford, MA, USA). 

### 2.2. Samples Preparation and Extraction

The MEPS methodology was selected for the extraction of analytes from oral fluid samples and calibration curves. MEPS consists of the miniaturization of conventional SPE with a conditioned needle incorporated directly into an injector syringe (Trajan Scientific Australia Pty Ltd) which is an alternative to other extraction methodologies in different matrixes [36], thus demonstrating its specificity and reduction in matrix effects [37,38], including in oral fluid samples [16,21,27,39,40]. 

In summary, a needle attached to the MEPS was conditioned with methanol (50  µL) and water (2  ×  50 µL) before the first use. Oral fluid samples with meloxicam-D3 (IS) were extracted using a syringe with an MEPS Barrel Insert and Needle (BIN) device. In this sense, when the samples passed through the solid phase (packed bed), the specific analytes were adsorbed. The solid phase was then washed with 50  µL of Milli-Q water to remove interferents and non-specific analytes. A solution of methanol and ammonium acetate 10 mM (80  v:20  v) was used for the elution (100  µL) of the adsorbed analytes. The solution with specific analytes was dispensed into a vial and injected (5 µL) into the LC-MS/MS device for analysis. Finally, the syringe and BIN were washed (100  µL—5× MeOH) between each patient sample. The same process was performed for the calibration curve [21,27,38].

The extraction steps with the MEPS device, which were standardized in previous works by our group [21,27], are described in Table 1.

### 2.3. Standard Solutions and Analytical Validation

Stock solutions of meloxicam (1 mg/mL methanol), 5′-carboxymeloxicam (1 mg/mL methanol), PGE_2_ (1 mg/mL methanol) and IS (1 mg/mL methanol) were prepared. A dilution series from each stock solution (10 ng/mL) was used to construct standard curves. Solutions were stored in the dark at −20 °C until use, and all stages of the research were conducted under a sodium vapor lamp to avoid the photodecomposition of the analytes. 

The calibration curve for the oral fluid samples was prepared using the following concentrations: 625, 312.5, 156.2, 78.1, 39.1, 19.5, 9.8, 2.4, 1.22 and 0.61 ng/mL for meloxicam and 5′-carboxymeloxicam. For the PGE_2_ calibration curve, the concentrations used were: 2500, 1250, 625, 312.5, 156.2, 78.1, 39.1, 19.5, 9.8 and 2.4 ng/mL. All polypropylene tubes were stored at −20 °C until use [21,27,28,31].

The method of analysis of meloxicam and its main metabolite, 5′-carboxymeloxicam, was validated in accordance with the United States Food and Drug Administration (US FDA) recommendations. Industry guidance: bioanalytical method validation [41]. 

Quality controls (QCs) for the oral fluid were prepared by adding the standard solutions to blank oral fluid. The parameters evaluated were linearity, accuracy, precision, medium-quality control (MQC), high-quality control (HQC), lower limits of quantification (LLOQ), low-quality control (LQC), stability and dilution.

The matrix effect was evaluated by comparing the peak areas obtained from meloxicam, 5′-carboxymeloxicam and IS directly in the mobile phase (no matrix) with the peak in the presence of a matrix (blanks added to standard solutions of meloxicam, 5′-carboxymeloxicam and IS after extraction). The IS matrix normalization factor was calculated for each sample matrix by dividing the analyte ratio by the IS response in the absence of the matrix. The coefficient of variation for the normalized IS had to be less than 15%. The samples were analyzed between the LQC and HQC.

Linearity was determined by three linear calibration curves for each analyte (meloxicam, 5′-carboxymeloxicam) using a 1/*χ*^2^ weighted equation linear mathematical model. The r2 value of all curves was calculated.

Accuracy (RE%) and precision (CV%) were determined by intra- and inter-assay performance (three different assays). Each was run with the LLOQ, LQC, MQC and HQC was repeated five times for each step. The acceptable range had to be between 15% and 20% of the nominal value.

The LLOQ is defined as the lowest quantified concentration. This parameter was determined by ten analyses of samples with 0.61 ng/mL of meloxicam, 5′-carboxymeloxicam and IS in the oral fluid. The interfering peaks close to the retention time of the studied analyte had to be less than 20%. The maximum allowable LLOQ had to be within 20% of the nominal value.

The stabilities of meloxicam and 5′-carboxymeloxicam in the oral fluid were evaluated using the LQC and HQC, analyzed three times after the preparation and stored under the following three conditions: (1) short-term (12 h at 23 °C), where the samples were evaluated after being kept at 23 °C for 12 h; (2) post-processing (12 h at 4 °C), where the samples were kept at 4 °C for 12 h at the autoinjector temperature and (3) freeze/thaw cycle (−70 °C), where the samples were frozen at −70 °C for at least 12 h and thawed at 25 °C for 60 min. After three cycles of freezing and thawing, the samples were analyzed. All samples were analyzed using freshly prepared calibration curves. The sample data were considered stable when the concentrations were within 15% of the nominal value [21,27,30,31].

For the calibration curves, a comparison of the chromatograms obtained from samples enriched with a standard and a blank (non-enriched) was performed. Therefore, it was possible to establish the selectivity and interference of the other components of the samples [21,31,42].

### 2.4. LC-MS/MS

The concentrations of meloxicam, its main metabolite, 5′-carboxymeloxicam, PGE_2_ and meloxicam D-3 (internal standard—IS) were detected and quantified using a Triple Quadrupole 8040 Mass Spectrometer (Shimadzu, Kyoto, Japan). Drug characterization was performed using LC-MS/MS, and separation was performed using a Shim-Pack XR-ODS 75 L × 2.0 column and a C18 pre-column (Shimadzu, Kyoto, Japan) at 40 °C with a mixture of methanol and 10 mM ammonium acetate (80:20, *v*/*v*) with an injection flow rate of 0.3 mL/min. The total analytical run time was 5 min.

Detection and quantification in the oral fluid samples was performed after optimizing the analytes in the multiple reaction monitoring (MRM) mode in the quantitative analysis. The technical conditions of the equipment used were as follows: the electrospray ionization source (ESI) had a voltage of 4.5 kV; the source temperature was maintained at 250 °C; the desolvation temperature was maintained at 350 °C; the collision gas was argon gas (230 kPa); the mist gas was nitrogen (3.0 L/min). The cone voltage was set for each transition, and specific analyte ions were fragmented. The specific conditions for analysis and quantification were initiated by the direct injection of standard drug solutions of meloxicam, 5′-carboxymeloxicam and PGE_2_ at a concentration of 10 ng/mL and 1 ng/mL for the IS solution without the separation column; thus, the precursors and products (*m*/*z* = mass number/charge number) of the specific analytes were obtained. All parameters found for the analytes are shown in Table 2.

Data acquisition and sample quantification were performed using the LabSolutions software, version 5.97 (Shimadzu, Kyoto, Japan).

### 2.5. Volunteers and Sample Collection for PK Analysis

Sixteen volunteers were invited for this study. All volunteers fasted for at least 2 h. They received a 15 mg meloxicam tablet and seventeen 50 mL falcon tubes, which were duly identified with the following collection times: before receiving the tablet; 0.25; 0.5; 0.75; 1; 1.5; 2; 3; 4; 5; 6; 8; 11; 24; 48; 72 and 96 h after taking the tablet. The volunteers were instructed to collect a sample of oral fluid of approximately 4 mL at each collection time and store it in a refrigerator in the box provided by the research team. After delivering the samples, they were centrifuged for 10 min (2500 rpm), and the supernatant was stored at −20 °C until analysis.

The pharmacokinetic data of meloxicam and its main metabolite, 5′-carboxymeloxicam, were estimated in the oral fluid for the following defined parameters:

(a) Area under the curve from zero to the last quantifiable time (AUC0-t);
(b) Expected total clearance (Cl/f);
(c) Volume of distribution (Vd/F);
(d) Drug elimination half-life (t1/2);
(e) Elimination constant (Kel);
(f) (Tmax);
(g) Estimated maximum observed concentration value (Cmax).

Through the concentrations obtained experimentally, the pharmacokinetic parameters were determined using the Phoenix WinNonlin Software (version 8.1) (Certara L.P, Princeton, NJ, USA.) with the non-compartmental model with elimination [27,28,31].

### 2.6. Statistical Analysis

All samples from each participant were analyzed at the same time. After quantifying the oral fluid concentrations of meloxicam, 5′-carboxymeloxicam and PGE_2_ by LC-MS/MS, the data were submitted for statistical analysis using the Jamovi Software (version 2.3.9) and organized in Sigmaplot (version 14.0) for graphics. The Friedman test followed by the Durbin–Conover multiple comparisons test was performed for the three analytes studied. The significance level adopted was 5%. Pharmacokinetic parameters are presented as mean and standard deviation (SD).

## 3. Results

The meloxicam, 5′-carboxymeloxicam and PGE_2_ concentrations in the oral fluid were determined and quantified from the oral fluid samples collected from Caucasian volunteers before and up to 96 h after a single oral dose of meloxicam (15 mg). The descriptive data of the sixteen volunteers are presented in Table 3.

### 3.1. Calibration Curve of Meloxicam and 5′-Carboxymeloxicam and PGE_2_

Calibration curves with known standard concentrations were added to blank oral fluid samples to perform the concentration analyses on the volunteer samples.

**Figure 2:**
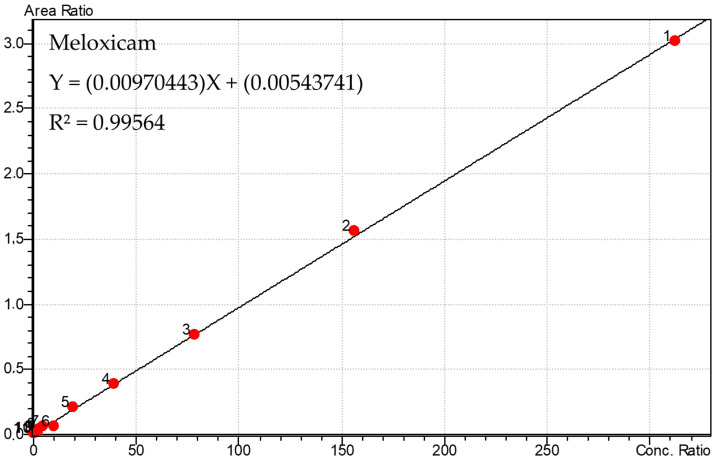
Calibration curve used for quantification of meloxicam in oral fluid samples with points from 1 to 10, respectively. (625, 312.5, 156.2, 78.1, 39.1, 19.5, 9.8, 2.4, 1.22 and 0.61 ng/mL).

**Figure 3:**
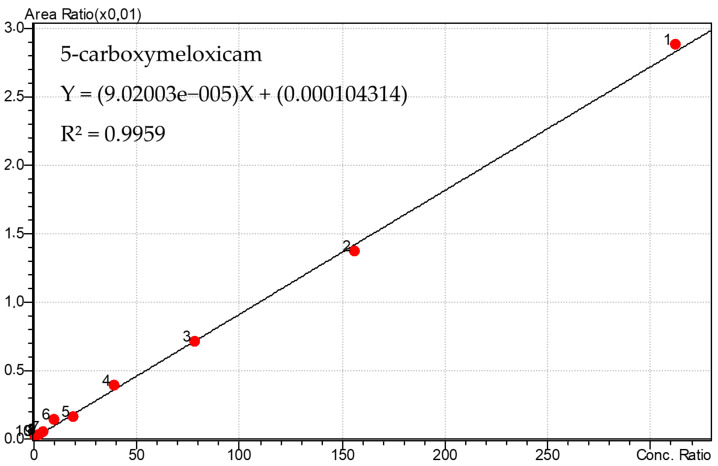
Calibration curve used for quantification of 5′-carboxymeloxicam in oral fluid samples with points from 1 to 10, respectively. (625, 312.5, 156.2, 78.1, 39.1, 19.5, 9.8, 2.4, 1.22 and 0.61 ng/mL).

The calibration curves used are shown in Figure 2 and Figure 3. Considering the stability of the curves, the analyses of the samples were performed.

### 3.2. PK Analysis

For the detection of meloxicam and 5′-carboxymeloxicam in the oral fluid, the LLOQ was 0.6103 ng/mL, the LQC was 2.44 ng/mL, the MQC was 78.12 ng/mL and the HQC was 156.25 ng/mL. The DQC used 1250 ng/mL of meloxicam and 5′-carboxymeloxicam in the oral fluid.

PGE_2_ was detected in the oral fluid samples after the ingestion of a meloxicam tablet (15 mg) by the volunteers, demonstrating the possibility of a PK/PD study with this methodology.

**Figure 4:**
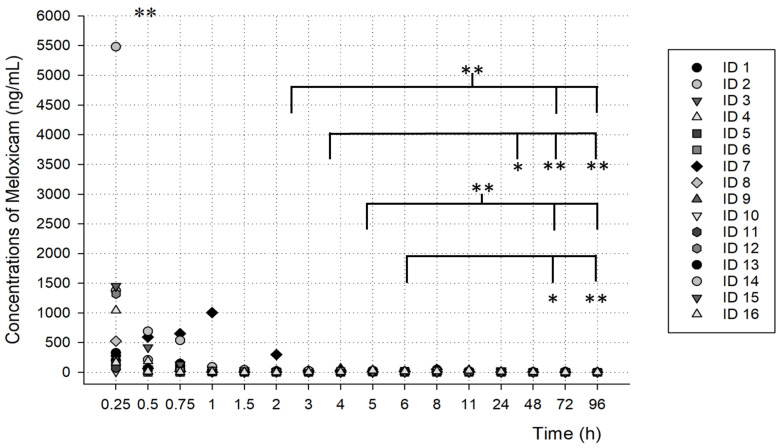
Meloxicam concentrations over time, analyzed in oral fluid samples from volunteers (statistically significant difference ** *p* < 0.001; * *p* < 0.05).

**Figure 5:**
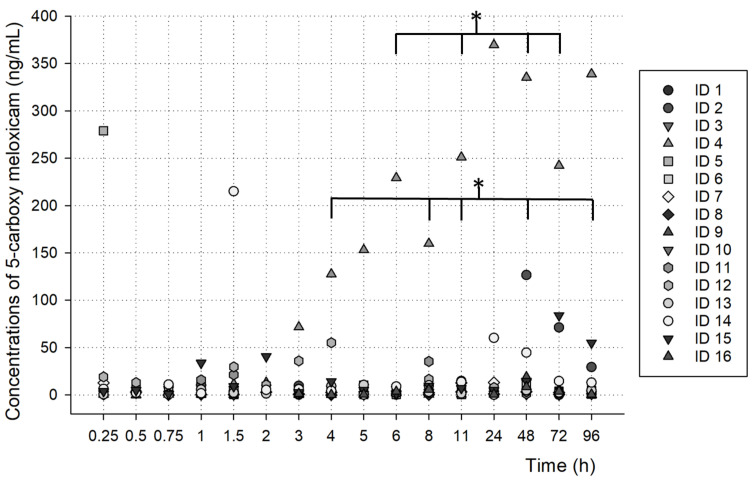
5′-carboxymeloxicam concentrations over time, analyzed in oral fluid samples from volunteers (statistically significant difference * *p* < 0.05).

As shown in Figure 4 and Figure 5, the concentrations found at each time after the oral administration of a meloxicam tablet (15 mg) demonstrated the possible metabolism profiles of meloxicam and 5′-carboxymeloxicam over time.

The concentrations obtained, as shown in Figure 4, were different between the times analyzed and showed a significant difference (** *p* < 0.001). When analyzing time by time, there was a significant difference between the samples at 4 h, 5 h, 6 h and 8 h and the samples at the final hours, mainly at 72 h and 96 h (* *p* < 0.05).

As shown in Figure 5, the 5′-carboxymeloxicam concentrations did not differ significantly over time. However, when evaluating the times individually, there was a significant difference between the samples at 4 h, 6 h, 8 h, 11 h, 48 h, 72 h and 96 h (*p* < 0.05).

### 3.3. PGE_2_ Analysis

**Figure 6:**
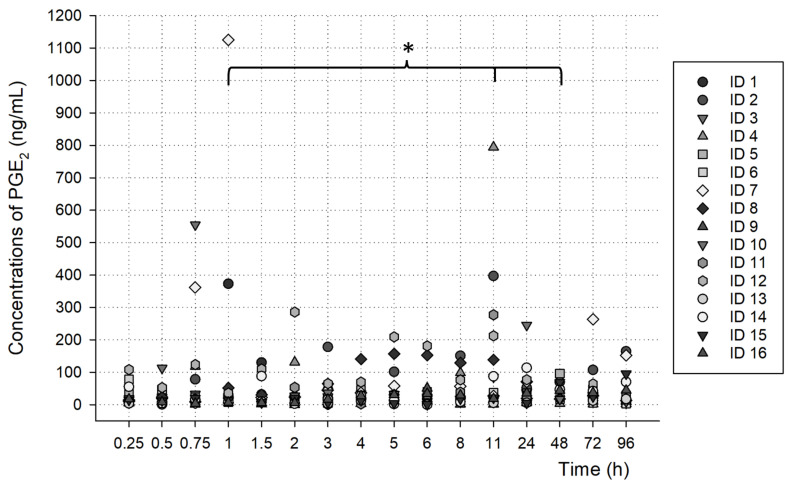
Oral fluid concentrations of PGE_2_ over time, analyzed after administration of 15 mg of meloxicam (* *p*  <  0.05).

The quantified concentrations of PGE_2_ of all the volunteers over time are shown in Figure 6. There was a significant difference in the concentrations obtained when we observed each analyzed time, especially at the times of 1 h, 11 h and 48 h, which showed a significant difference (* *p*  <  0.05).

Table 4 shows the PK parameters obtained from the concentrations of meloxicam and its main metabolite, 5′-carboxymeloxicam, found in the oral fluid samples analyzed. All parameters are shown as mean ± standard deviation.

### 3.4. Method Validation

The analytical validation parameters for the methods of meloxicam and 5′-carboxymeloxicam in the human oral fluid are presented in Table 5. The validation for the PGE_2_ analysis was documented in a previous study by our research group [21]. The coefficient of variation in the normalized IS was less than 15% for all the analytes. The matrix effect of all the oral fluid samples (*n* = 6) was absent for both meloxicam and its main metabolite, 5′-carboxymeloxicam. For the LQC = 2.44 ng/mL, the coefficient of variation in the IS normalized matrix factor was 7.9 and 12.67 for meloxicam and 5′-carboxymeloxicam, respectively. For the HQC = 156.25, the coefficient of variation in the IS normalized matrix factor was 9.34 and 11.5 for meloxicam and 5′-carboxymeloxicam, respectively.

The linearity was 0.6103 at 625 ng/mL, with r2 = 0.995, for meloxicam, and it was 0.6103 at 625 ng/mL, with r2 = 0.9959, for its main metabolite, 5′-carboxymeloxicam. Precision and accuracy had a coefficient of variation <15%, indicating an accurate and reproducible analysis. The intra- and inter-assay precision and accuracy, as represented by the coefficient of variation (CV) and relative error (RE), respectively, were <15% for both the analytes studied in the oral fluid.

Three freeze (−70 °C)/thaw (23 °C) cycles were performed, and meloxicam and its main metabolite, 5′-carboxymeloxicam, demonstrated stability in the oral fluid after 12 h at 23 °C and after sample post-processing up to 12 h at 4 °C, with deviations of less than 15%.

The quality control to achieve dilution integrity (DQC—1250 ng/mL; 1:5 dilution) of meloxicam and its main metabolite, 5′-carboxymeloxicam, showed a coefficient of variation of less than 15%

The validation parameters were evaluated with freshly prepared calibration curves. Stability was accepted when the deviation from the nominal value was equal to or less than ±15% [21,27,28,31].

## 4. Discussion

Recent works by our research group have demonstrated that the use of oral fluid samples for PK/PD studies is promising. The concentrations found in the oral fluid corresponded to the free or unbound fraction of several drugs, which is the parameter used to study the pharmacological or toxic action of a drug [26,27,28,29,30,31]. Considering it is easy to obtain oral fluid samples from volunteers [22,24,30,37], it is possible to predict that for meloxicam and its main metabolite, 5′-carboxymeloxicam, oral fluid samples can be considered an alternative method to blood samples. For PK/PD studies, for example, numerous collections are required to determine possible concentrations over time, which allows performing the calculations as needed for the parameters of interest. Therefore, if in the future we are able to extrapolate these findings, it may be possible to monitor and carry out a personalized prescription by assessing the individual metabolism, the genetic profile or the systemic conditions of patients that may affect the safety and efficacy of a treatment.

Meloxicam has a very peculiar latency time, as its packaging insert informs that the T_Max_ can vary between 5 and 6 h, a finding that corroborates the findings of Turck et al, who described the pharmacokinetic profile of the drug under study in plasma samples [2,4]. In the oral fluid samples, the values obtained were 1.41 ± 4.77 h, which were much higher than other NSAIDs already tested in this same fluid in our laboratory [27,28].

Meloxicam binds ~99.4% to human plasma proteins, mainly to albumin. The free fraction of the studied drug accumulates about 2.5 times more in synovial fluid than in plasma. After a single oral dose, the drug concentrations in synovial fluid may range from 40% to 50% of those found in plasma. Hence, it is expected that the concentrations found in saliva would be relatively lower and, consequently, some pharmacokinetic parameters obtained in this fluid may undergo significant variations related to this fact [4,43]. Therefore, despite the promising data from the present work, a study with a larger number of volunteers is needed.

In the results presented in this work, it was possible to observe a drop in the individual concentrations of meloxicam in the oral fluid samples over time. Shortly after this drop, a significant increase in the concentrations of its main metabolite, 5′-carboxymeloxicam, was observed over time. An overall decrease in PGE_2_ concentrations, despite the great individual variability, was also observed over time, which confirmed meloxicam’s effect by inhibiting COX-2 and, therefore, the importance of analyzing the influence of this important eicosanoid in inflammation studies.

The methodology developed in this research proved to be quite feasible, fast, sensitive and accurate. The use of MEPS proved to be effective and sensitive for the extraction of the specific analytes studied in this research in the oral fluid samples. Furthermore, the analysis through liquid chromatography-tandem mass spectrometry (LC-MS/MS) for the detection and quantification of different concentrations of meloxicam and its main metabolite, 5′-carboxymeloxicam, proved to be efficient and sensitive and corroborated similar results obtained in other studies by our group [21,27,28,31].

The time taken and the reduced use of plastic material were the other advantages of using the MEPS methodology. Compared to other extraction methodologies, MEPS proved to be faster. The entire extraction process with the instrument took less than 10 min, which was lower compared to other methods that can take more than 40 min to 1 h for each analysis. Due to the large number of samples from each volunteer required for a PK/PD study, the reduced time for the pre-analytical step is a relevant factor when choosing the extraction method [21,26,30,44,45,46]. Miniaturization is a new trend in the field of bioanalysis; thus, methods that consume less time, require less work and are more environmentally friendly [37,38,47] are more adequate.

The method presented here was validated according to the recommendations of the US FDA. Industry guidance: bioanalytical method validation (US FDA, 2018) [41]. All parameters evaluated in the validation of the methodology in the oral fluid samples proved to be stable and within the possible variations in each of the parameters described in this paper.

Despite the promising PK data obtained in this research, future works with a larger number of volunteers are necessary. Through the data presented here, we demonstrated the possibility of conducting a PK/PD study by detecting and quantifying meloxicam, its main metabolite and PGE_2_ in oral fluid samples using the methodology described.

## Figures and Tables

**Table 1:** Extraction method steps using MEPS.

MEPS Procedure Step	The Optimized Parameters
Conditioning	Methanol (100 μL) and water (50 μL)
Extraction	Draw-ejected, 100 μL of oral fluid
Washing	Milli-Q water (50 μL)
Elution	10 mM ammonium acetate + methanol (80 v: 20 v)
Washing solution	100 µL–Methanol

**Table 2:** Multiple reaction monitoring mode to quantify the meloxicam, 5′-carboxymeloxicam, PGE_2_ and meloxicam D-3 transitions.

Compound		Precursor (*m*/*z*)	Product (*m*/*z*)	RT (min)	Collision Energy (CE)	Q1 pre-Bias (V)	Q3 pre-Bias (V)	Dwell Time
Meloxicam	negative	350.20	286.05	0.886	14.0	27	19	21
			146.25		23.0	17	13	
5′-carboxymeloxicam	positive	381.9	144.95	0.722	−20	−14	−27	21
			170.95		−22	−19	−18	
Meloxicam-D3 (IS)	negative	353.10	289.25 149.20	0.888	14 20	14	19 15	21
Prostaglandin E2	negative	351.40	271.20	0.949	18	27	12	21
			315.20		12		21	

**Table 3:** Descriptive data of the sixteen volunteers.

Female (*n*)	Male (*n*)	Age—Years (Mean ± SD)	Body Weight—kg (Mean ± SD)	Height—m (Mean ± SD)
12	4	31.4 ± 9.9	75.8 ± 21.5	1.7 ± 0.1

**Table 4:** Pharmacokinetic parameters of meloxicam and its main metabolite, 5′-carboxymeloxicam, in oral fluid samples.

Mean ± SD		
PK Parameters	Meloxicam	5′-Carboxymeloxicam
AUC0-t (h× ng/mL)	532.26 ± 815.55	134,733.7 ± 350,579.04
Cl/F (L/h)	101,938.84 ± 102,796.59	48,055.38 ± 61,627.98
Cmax (ng/mL)	2145.22 ± 4220.51	105.07 ± 233.15
Kel (1/h)	31.57 ± 20.81	4.95 ± 10.45
T_1/2_ (h)	2.48 ± 6.89	47.26 ± 144.04
Tmax (h)	1.41 ± 4.77	81.76 ± 236.18
Vd/F (L)	167.55 ± 415.67	21,065.43 ± 3398.62

T_max_ and C_max_: time and value of the maximum observed concentration, respectively; AUC_0-t_: area under concentration versus time curve from the first observed concentration to the last one; Vd/F: estimated volume of distribution in total AUC; Clt/F: full clearance; Kel: elimination rate constant estimated from the regression line representing the terminal phase of the concentration–time profile; T_1/2_: terminal half-life of the drug.

**Table 5:** Analytical validation of parameters for the methods of meloxicam and 5′-carboxymeloxicam in human oral fluid.

	Meloxicam		5′-Carboxymeloxicam	
**Linearity**				
***r*** **^2^**	0.99564		0.9959	
**Equation of the line**	Y = (0.00970443) X + (0.00543741)		Y = (9.02003 × 10^−5^) X + (0.000104314)	
**Low-quality control (ng/mL)**	2.44		2.44	
**Precision (CV%; *n* = 10)**	7.98		13.99	
**Accuracy (%)**	−2.37		0.4	
**Precision (CV%) and Accuracy (RE%)**				
***Intra-assay (n = 3)***	CV	RE	CV	RE
LLOQ (0.6103 ng/mL)	9.69	−0.38	9.81	5.63
LQC (2.44 ng/mL)	8.78	−3.63	13.17	5.41
MQC (78.12 ng/mL)	11.63	−9.67	6.09	4.11
HQC (156.25 ng/mL)	13.17	12.19	12.64	6.86
DQC (1250 ng/mL; 1:5)	12.02	−1.17	12.56	−6.07
***Inter-assay (n = 8)***				
LLOQ (0.6103 ng/mL)	11.38	7.43	10.84	10.87
LQC (2.44 ng/mL)	8.06	9.32	11.87	−2.77
MQC (78.12 ng/mL)	13.88	−6.32	5.79	−3.18
HQC (156.25 ng/mL)	4.75	0.59	6.02	−1.7
**Stabilities (*n* = 3)**				
***Short-term stability (12 h at 23 °C)***				
LQC (2.44 ng/mL)	9.8		3.78	
HQC (156.25 ng/mL)	11.6		11.82	
***Post-processing stability (12 h at 4 °C)***				
LQC (2.44 ng/mL)	13.04		7.8	
HQC (156.25 ng/mL)	8.3		13.99	
***Freeze/thaw cycle stability (−70 °C)***				
LQC (2.44 ng/mL)	−8.7		−6.53	
HQC (156.25 ng/mL)	5.2		10.25	

CV: coefficient of variation ((standard deviation/mean) × 100); r: linear correlation coefficient; RE: relative error ((observed concentration—nominal concentration)/nominal concentration) × 100; LLOQ: lower limit of quantification; LQC: low-quality control; MQC: medium-quality control; HQC: high-quality control; DQC: quality control for dilution integrity.

## Data Availability

The data presented in this study are available in the article.
